# Supplementary material for: Forchlorfenuron-Induced Mitochondrial Respiration Inhibition and Metabolic Shifts in Endometrial Cancer
Source: Cancers (Basel). 2024 Feb 28;16(5):976. doi: 10.3390/cancers16050976 (PMC10931130; doi:10.3390/cancers16050976)
Supplement: Supplementary file 1 [file cancers-16-00976-s001.zip › cancers-2846712 Figures S1-S6.pdf]

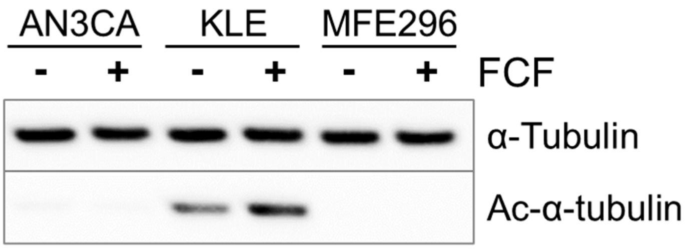

Figure S1. FCF increased tubulin acetylation in KLE cells: Three different endometrial cancer cell lines, AN3CA, KLE, and MFE296 were treated with FCF (100 μM, 48 h). Protein expression was determined by immunoblotting using specific antibodies as indicated.

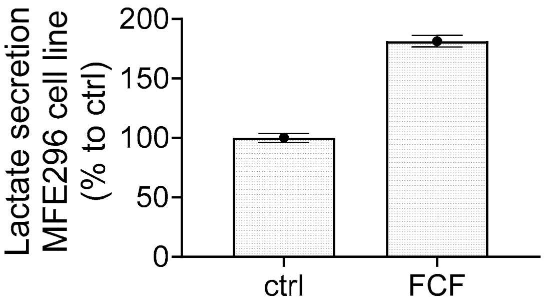

Figure S2. FCF enhanced lactate secretion MFE296 cells. MFE296 cells were treated with FCF (100 μM) for 24 hours. The concentration of lactate in supernatants was measured by enzyme-based lactate oxidation as described in “Materials and Methods” and normalized to protein levels of each sample (N=2).

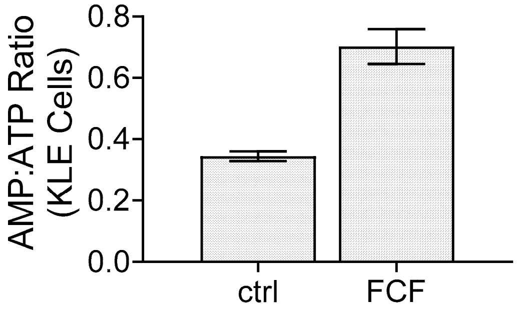

Figure S3. FCF led to an increase in the AMP to ATP ratio in KLE cells. KLE cells were treated with DMSO or FCF (100 μM) for 6 hours. Cell extracts were analyzed for ATP and AMP levels as described in "Materials and Methods".

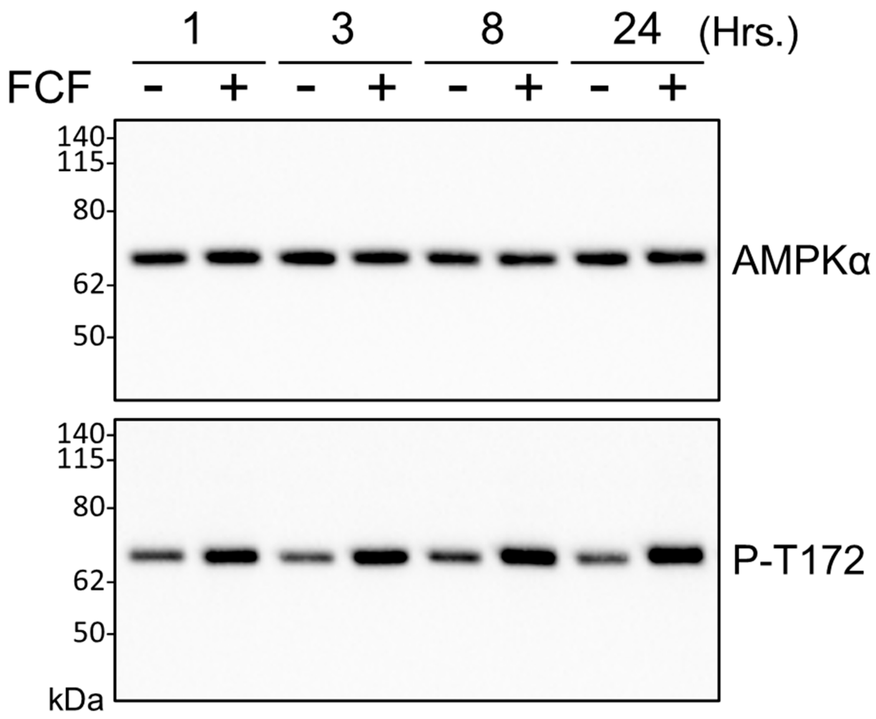

Figure S4. FCF activated AMPK in KLE cells. Immunoblotting was performed to measure total or phosphorylated AMPK (T172).

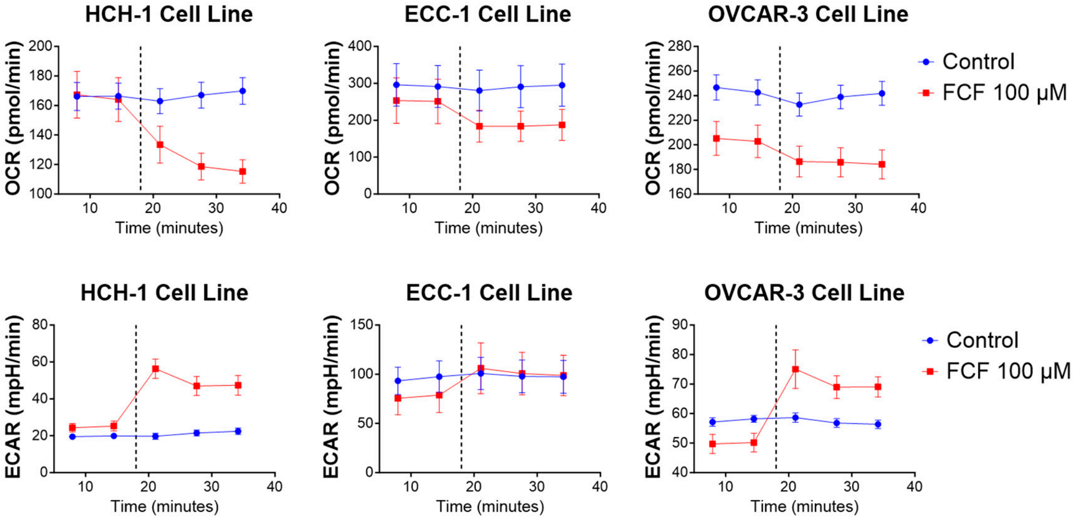

Figure S5. FCF inhibited the mitochondrial respiration of various cancer cell lines. The effects of FCF on the oxygen consumption rate (OCR) and extracellular acidification rate (ECAR) were evaluated in HCH-1 ovarian, ECC-1 endometrial, and OVCAR-3 ovarian cancer cell lines. The experimental procedures for OCR and ECAR measurements were described in the 'Materials and Methods' section. The dotted line represents the point of drug injection.

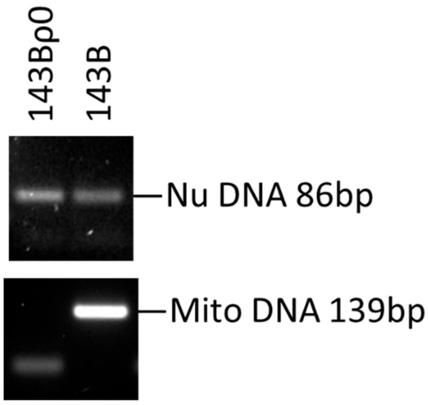

Figure S6. The depletion of mitochondrial DNA in 143Bρ0 cells: PCR amplification was conducted using a T100 thermal cycler (Bio-Rad) with the following primers: Nuclear DNA, B2 microglobulin (sense: TGC TGT CTC CAT GTT TGA TGT ATC T, antisense: TCT CTG CTC CCC ACC TCT AAG T); mitochondrial DNA, tRNA-Leu (sense: GAT GGC AGA GCC CGG TAA TCG C, antisense: TAA GCA TTA GGA ATG CCA TTG CG).
