# Supplementary material for: Forchlorfenuron-Induced Mitochondrial Respiration Inhibition and Metabolic Shifts in Endometrial Cancer
Source: Cancers (Basel). 2024 Feb 28;16(5):976. doi: 10.3390/cancers16050976 (PMC10931130; doi:10.3390/cancers16050976)
Supplement: Supplementary file 1 [file cancers-16-00976-s001.zip › cancers-2846712 File S1-Original Western Blot Images.pdf]

Figure 2G

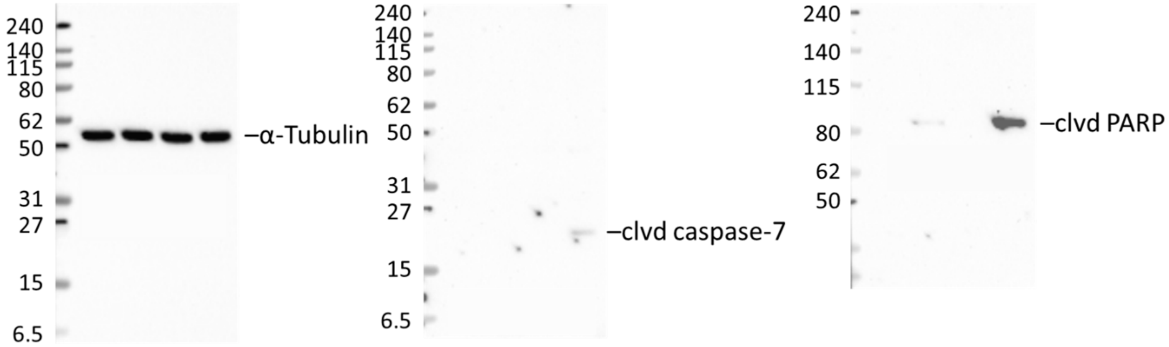

Figure 3A

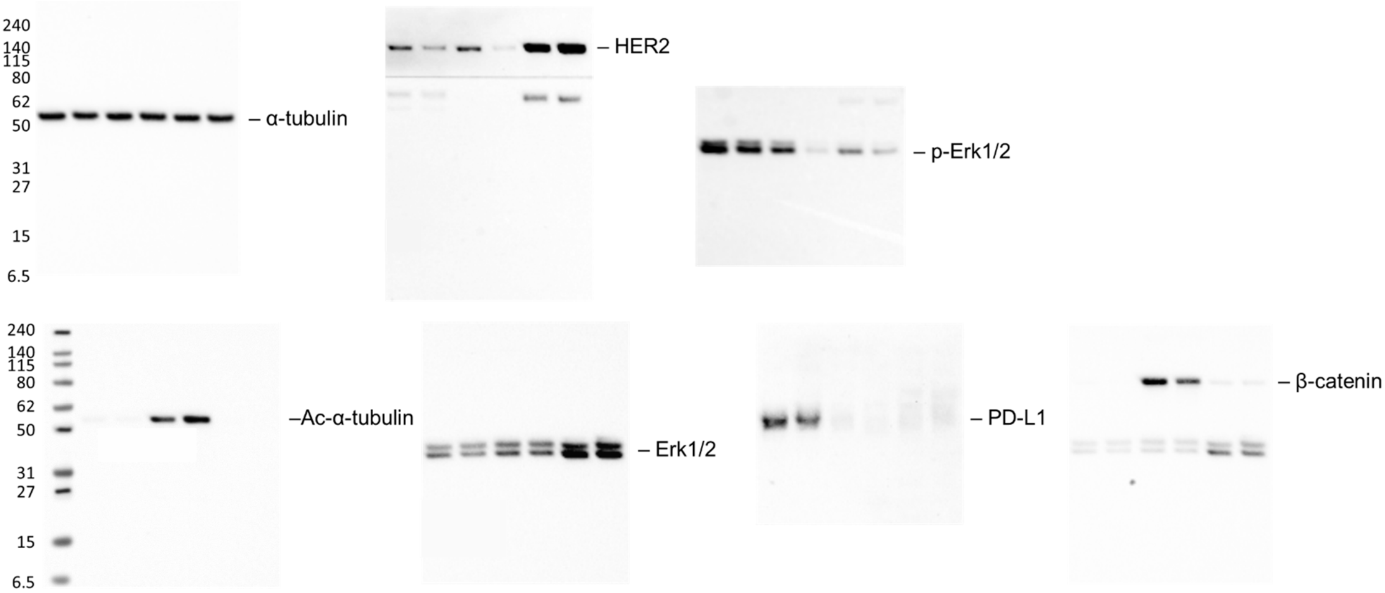

Figure 3B

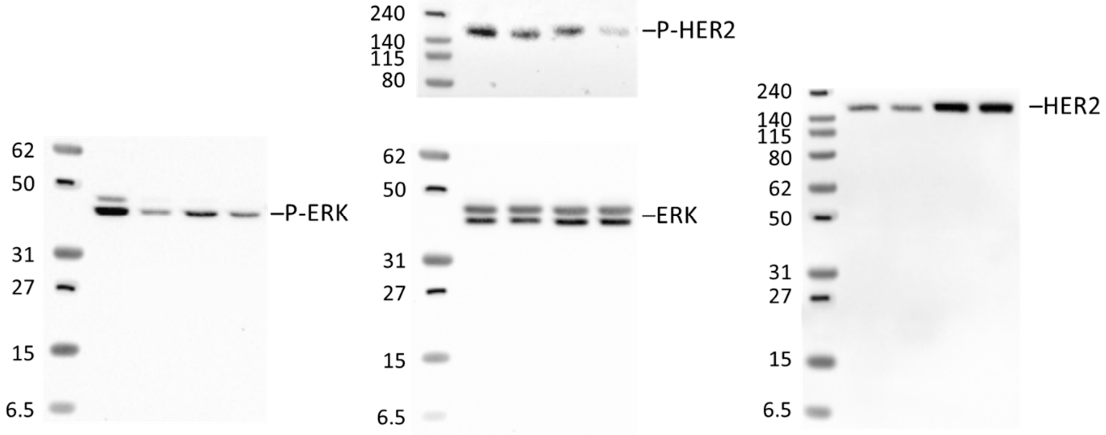

Figure 3D

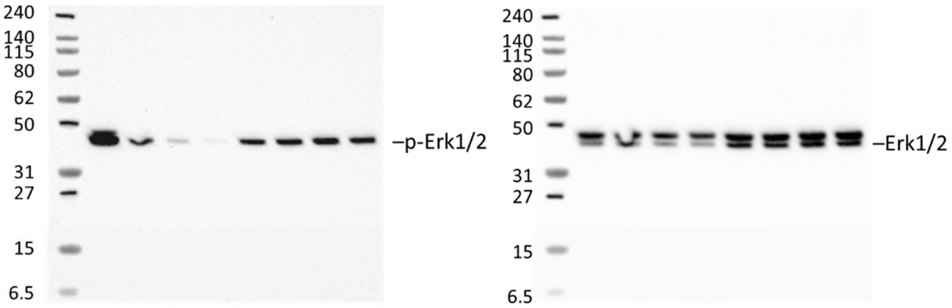

KLE	MFE296	Lapatinib (µM)
Lane 1	Lane 5	0
Lane 2	Lane 6	0.1
Lane 3	Lane 7	1
Lane 4	Lane 8	10

Figure 3E

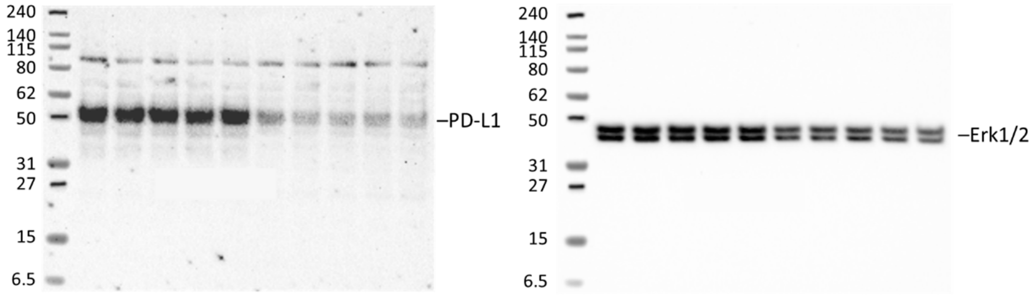

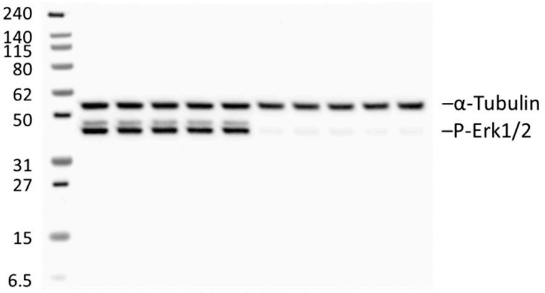

Lane	
1-5	ctrl
6-10	FCF 100

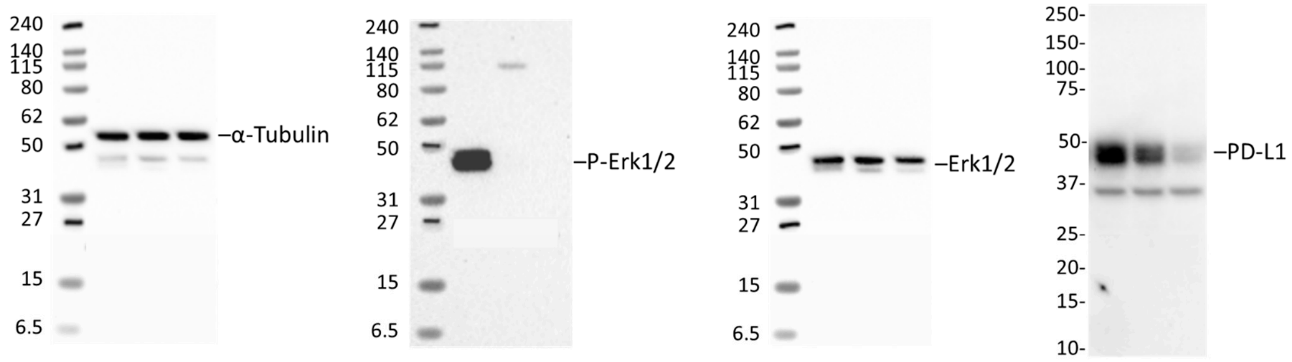

Lane 1	ctrl	
Lane 2	5	CI-1040 (μM)
Lane 3	20	

Figure 4F

Lane#		24 h
1	5	ctrl
2	6	2-DG 12 mM
3	7	FCF 100
4	8	2-DG+FCF

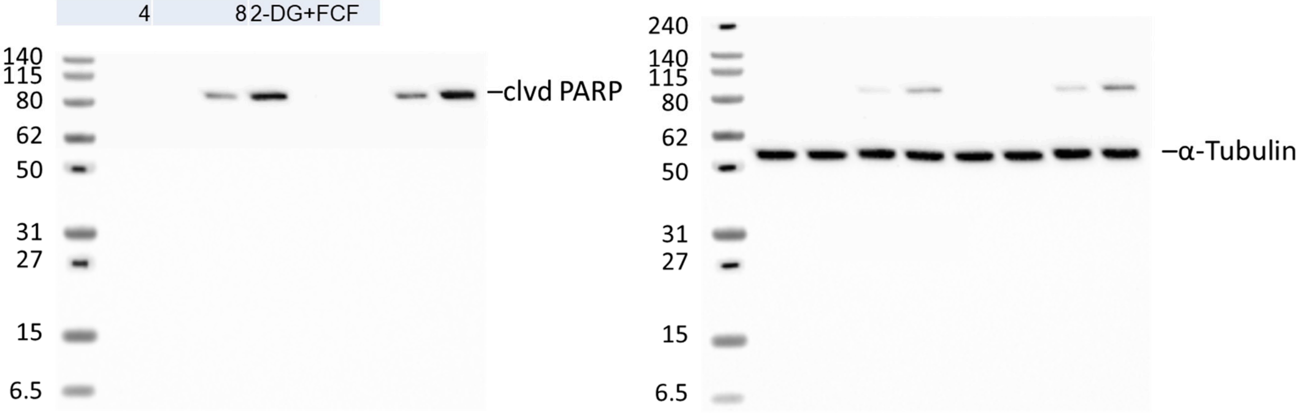

Lane #				24 h	
1	2	3	4	5	No Glu+FCF 100
6	7	8	9	10	Glu 1.0+FCF 100

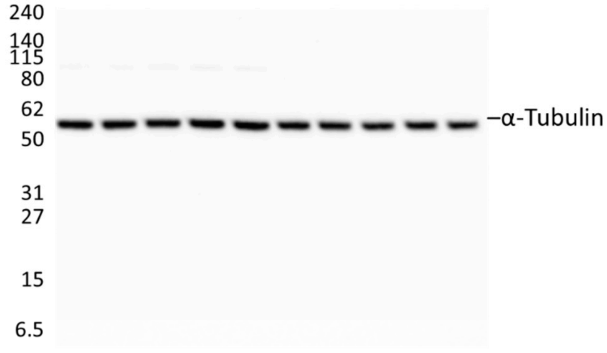

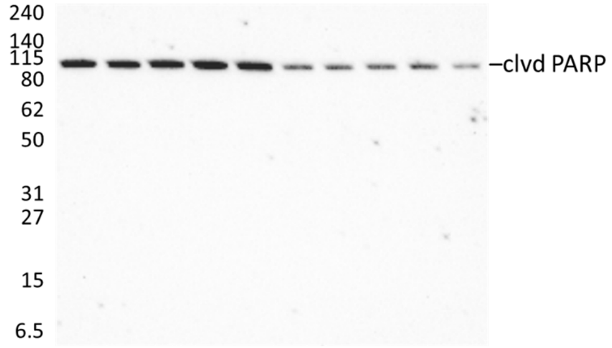

Figure 5A

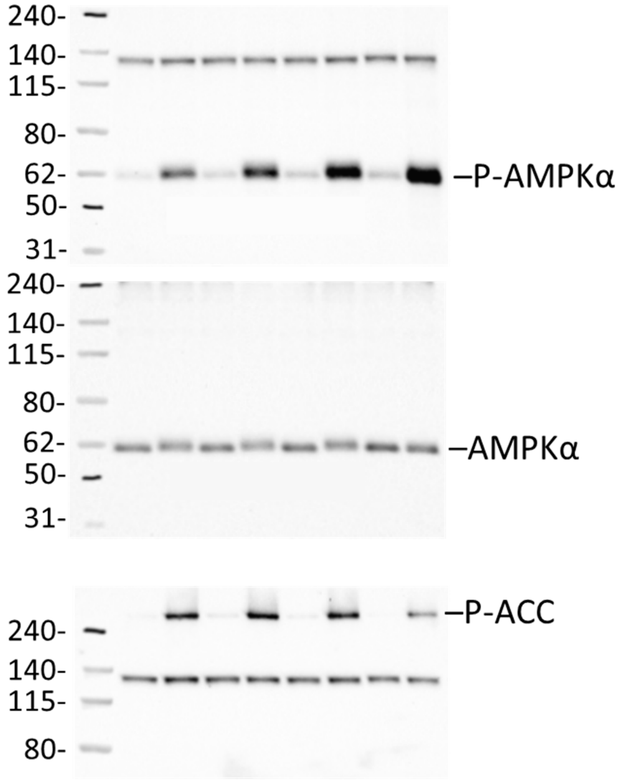

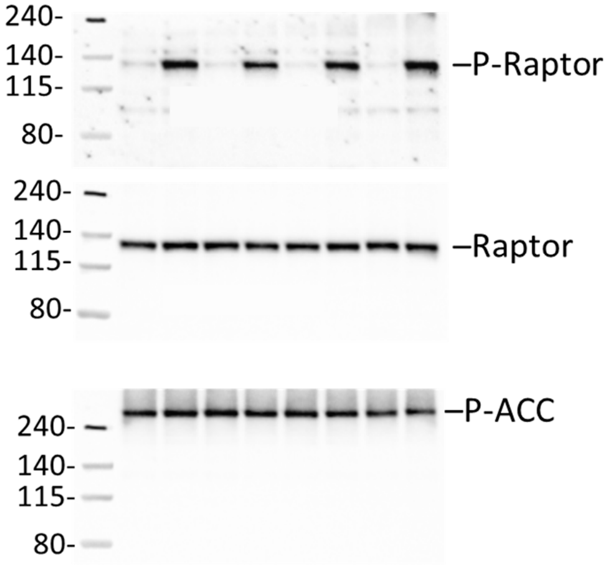

Figure 5B

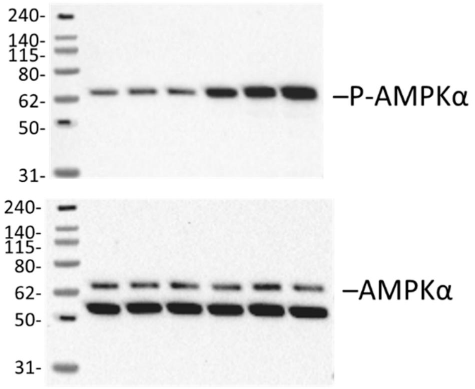

Figure 5C

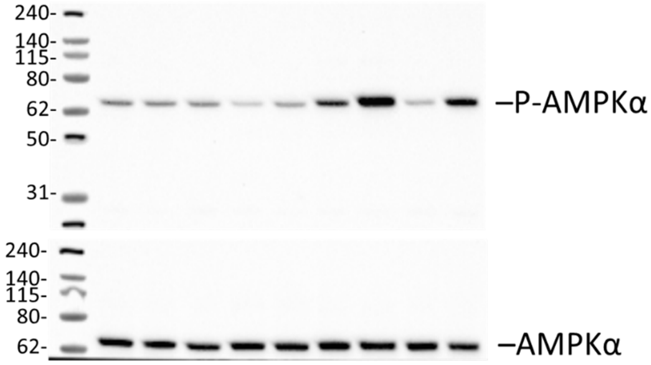

Figure 5F

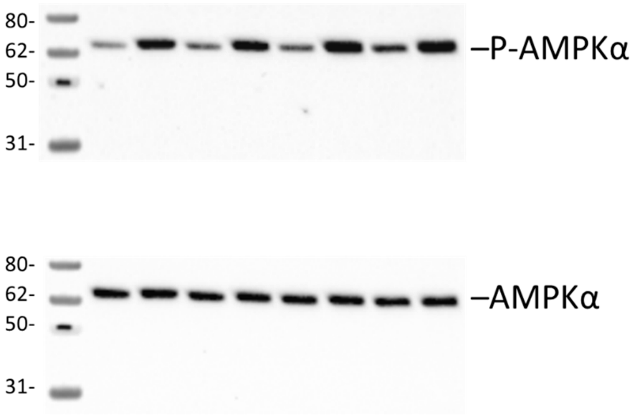

Figure 6A

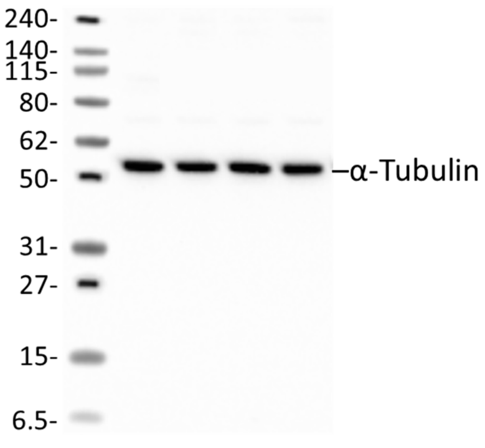

Figure 6C

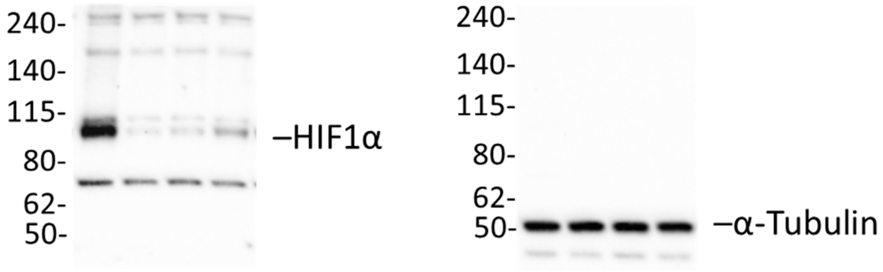

Figure 6F

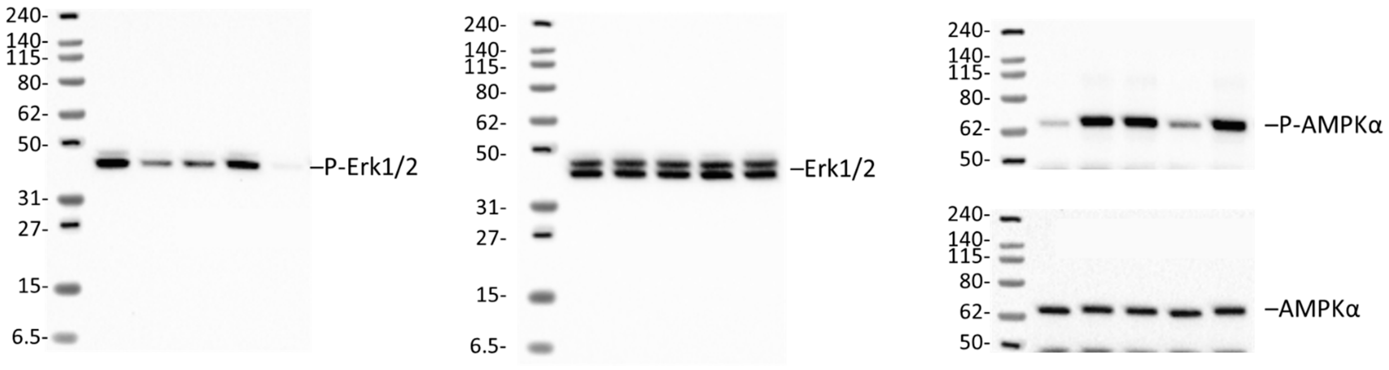

Figure 6G

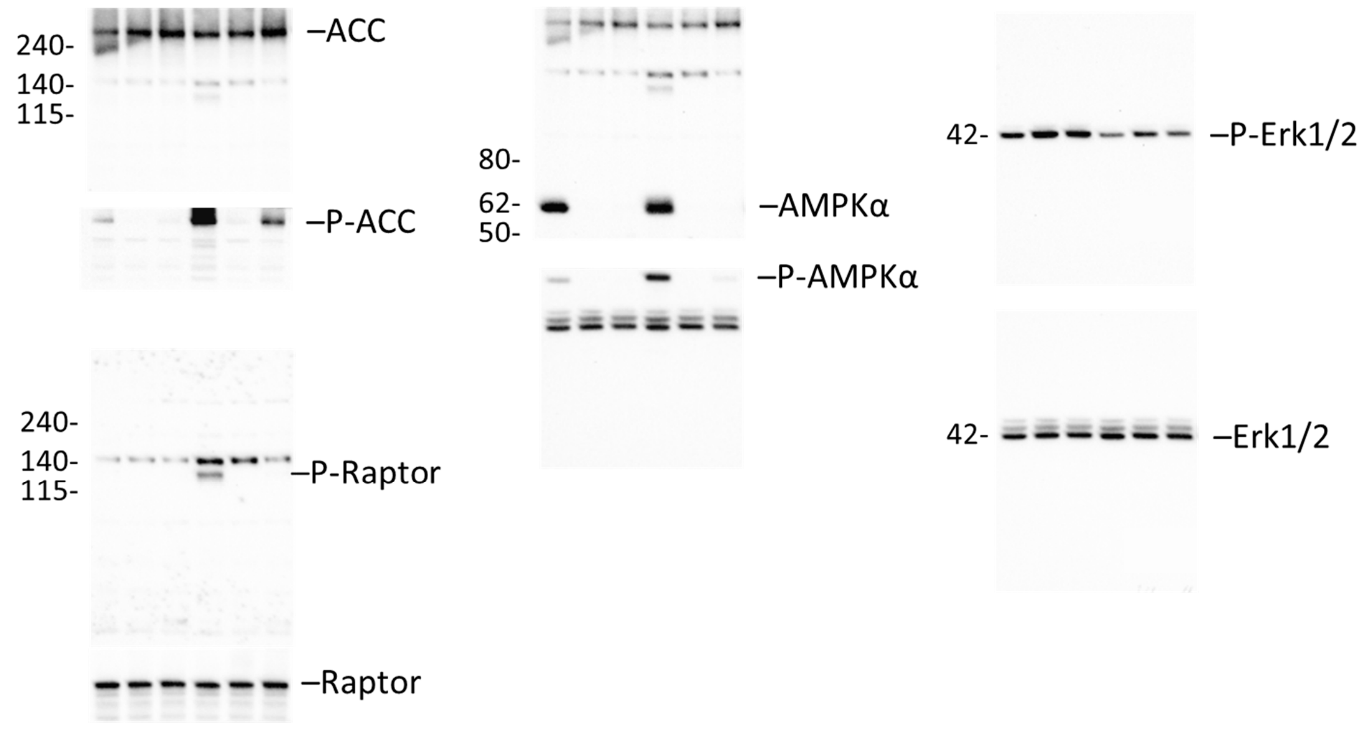

Figure 6H

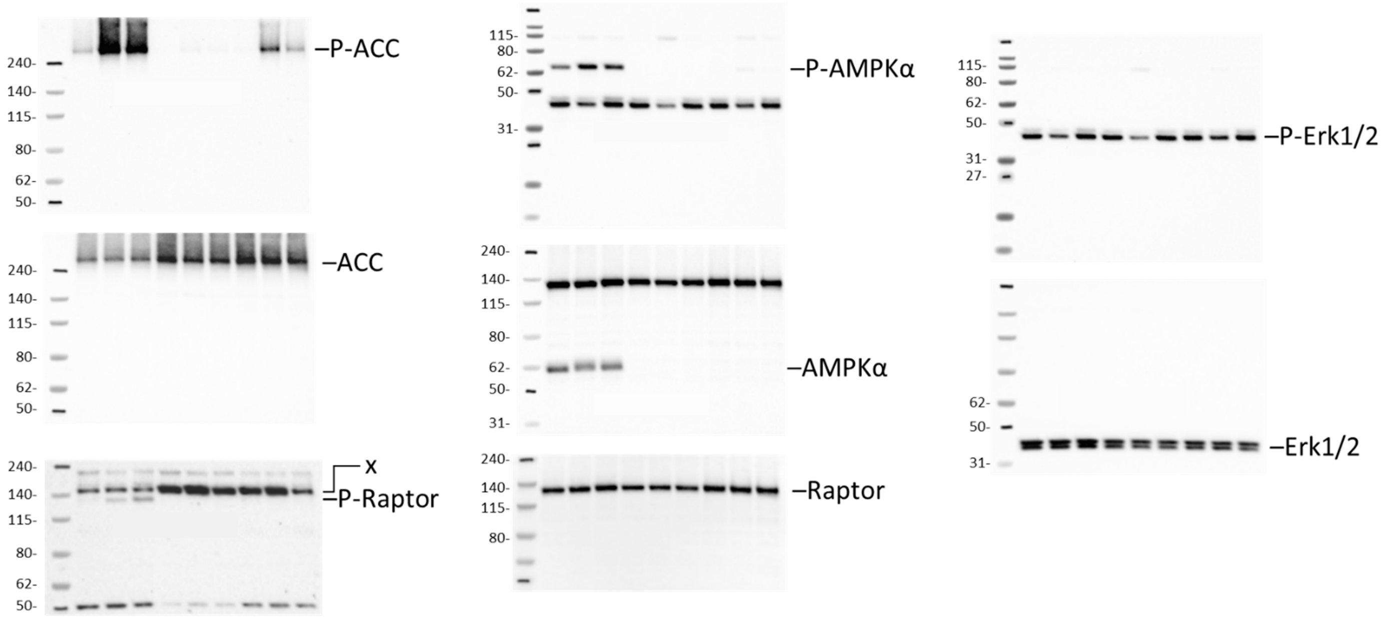

Figure 7A

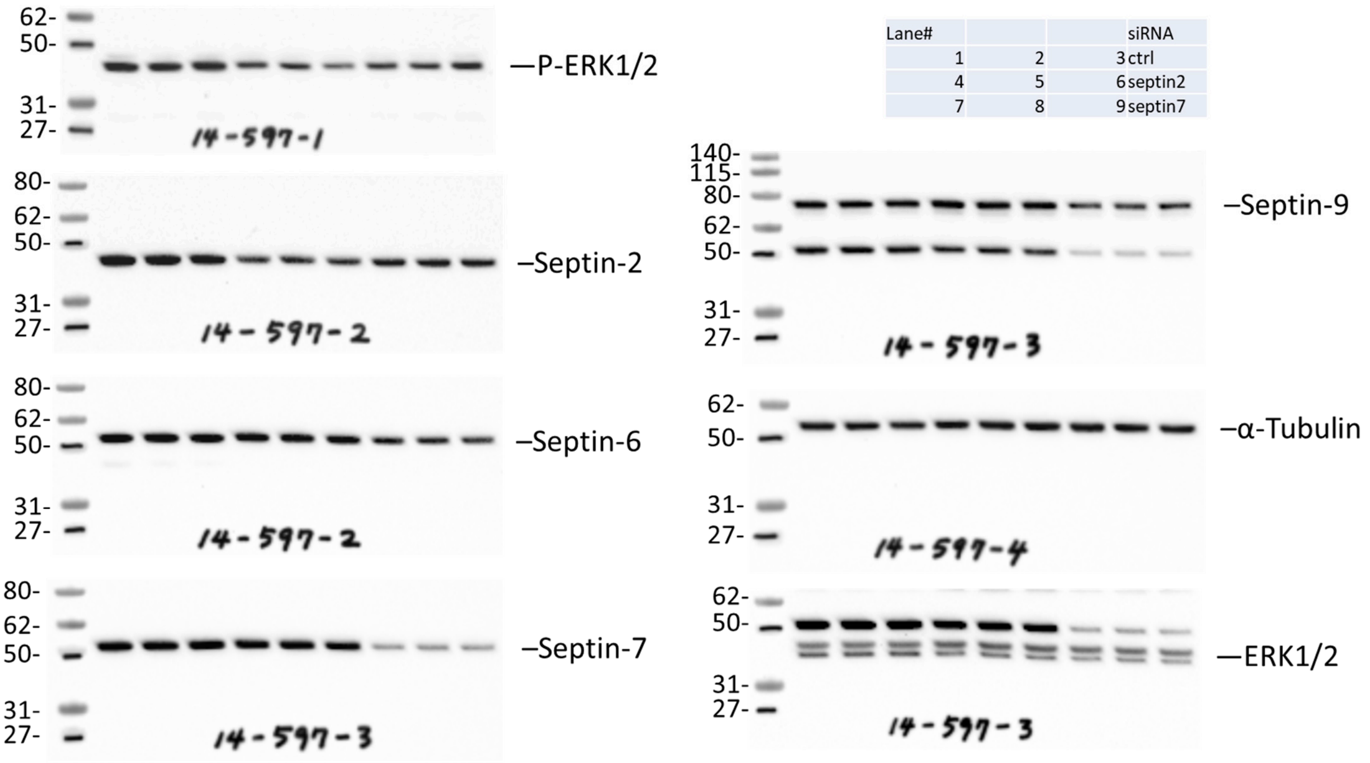

Lane#			siRNA
1	2	3	ctrl
4	5	6	septin2
7	8	9	septin7

Figure 7B

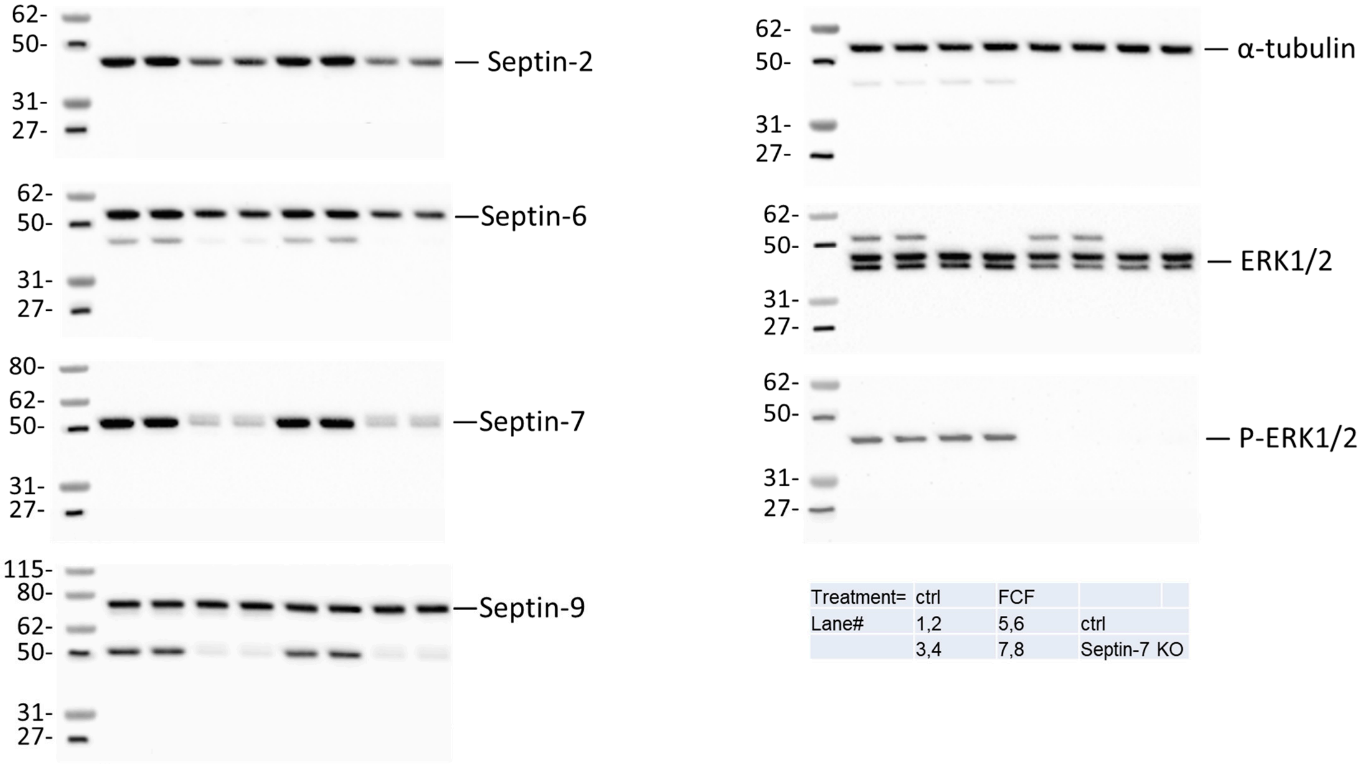

Treatment=	ctrl	FCF	
Lane#	1,2	5,6	ctrl
	3,4	7,8	Septin-7 KO

Figure 7C

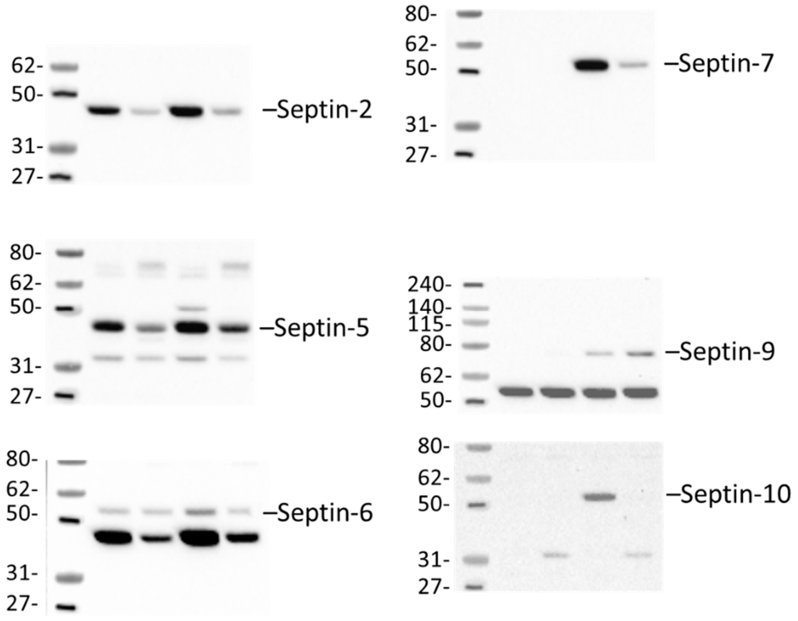
62-
50-
31-
27-
–Septin-2
80-
62-
50-
31-
27-
–Septin-7
62-
50-
31-
27-
–Septin-11
80-
62-
50-
31-
27-
–Septin-5
240-
140-
115-
80-
62-
50-
–Septin-9
80-
62-
50-
31-
27-
–α-Tubulin
80-
62-
50-
31-
27-
–Septin-6
80-
62-
50-
31-
27-
–Septin-10
